# A critical assessment of sympathetic restraint in submaximal exercise: Implications for integrated cardiovascular circuit control in exercise

**DOI:** 10.1113/EP091436

**Published:** 2025-03-25

**Authors:** Patrick J. Drouin, Stacey P. A. Forbes, Abby K. Zedic, Stuart P. S. Mladen, Michael E. Tschakovsky

**Affiliations:** ^1^ Human Vascular Control Laboratory, School of Kinesiology and Health Studies Queen's University Kingston Ontario Canada

**Keywords:** blood pressure, exercising muscle blood flow, oxygen delivery, sympathetic activity

## Abstract

Sympathetic restraint in exercising muscle is currently viewed as required to prevent ‘excess’ vasodilatation from exceeding the cardiac output ($\dot{Q}$) response, even in submaximal exercise. Certainly, muscle vasodilatory capacity dictates the requirement for sympathetic restraint when cardiac pumping capacity is approached. However, a similar role in submaximal exercise has at least two important implications for integrated cardiovascular control in exercise that have not been considered. First, such a role means that there is a ‘set’ $\dot{Q}$ response to a given exercise challenge that dictates the cardiovascular circuit flow and therefore the vasodilatation allowed such that $\dot{Q}$–peripheral blood flow balance and target arterial blood pressure are achieved. This represents a ‘cardiocentric’ model of integrated cardiovascular control, whereby the heart leads and the peripheral resistance vessel tone is modulated accordingly. Second, what is commonly described as ‘tight’ matching of exercising muscle oxygen delivery relative to demand would therefore require that the $\dot{Q}$ response is closely ‘calibrated’ to exercising muscle metabolic demand. This would require a means of driving cardiac activation via precise communication of exercising muscle metabolic demand. However, considerable evidence demonstrates that ‘excess’ vasodilatation in a healthy system simply leads to a matching increased $\dot{Q}$ without arterial blood pressure compromise. This review re‐examines the evidence for existence of sympathetic restraint in exercising muscle and its currently proposed role. We propose that key questions remain unanswered and that renewed investigation into sympathetic restraint and its role can lead to important advances in understanding integrated cardiovascular control in exercise.

## Abstract (highlights)

- **What is the topic of this review?**
  This review examines the current proposed role of sympathetic restraint in exercising muscle, i.e., to prevent ‘excess’ vasodilatation from exceeding the cardiac output response, even in submaximal exercise.
- **What advances does it highlight?**
  This review points out two implications of the current proposed role of sympathetic restraint that have been ignored. It also identifies important evidence suggesting that the current view might be incorrect.

## 1 INTRODUCTION

With the onset of large muscle mass exercise, increased metabolic demand in exercising muscle requires support from the cardiovascular system in the form of increased perfusion. The cardiovascular response is characterized by vasodilatory signals rapidly and substantially increasing vasodilatation of resistance vessels in the exercising muscle to allow the increase in perfusion (MacDonald et al., 1998; Mortensen et al., 2005) and a closely matched increase in cardiac output ($\dot{Q}$) (De Cort et al., 1991). Although there can sometimes be an early and transient drop in arterial blood pressure, indicating a brief mismatch between the increase in peripheral blood flow (PBF) and $\dot{Q}$ during the first few seconds of exercise, arterial blood pressure quickly recovers, indicating a rapid adjustment in $\dot{Q}$–PBF balance (Barbosa et al., 2015; Wieling et al., 1996). Ultimately, a new steady‐state cardiovascular circuit flow is reached, which meets exercising muscle metabolic demand (Nyberg & Jones, 2022) while achieving a target arterial blood pressure (Raven et al., 2006).

**FIGURE 1:**
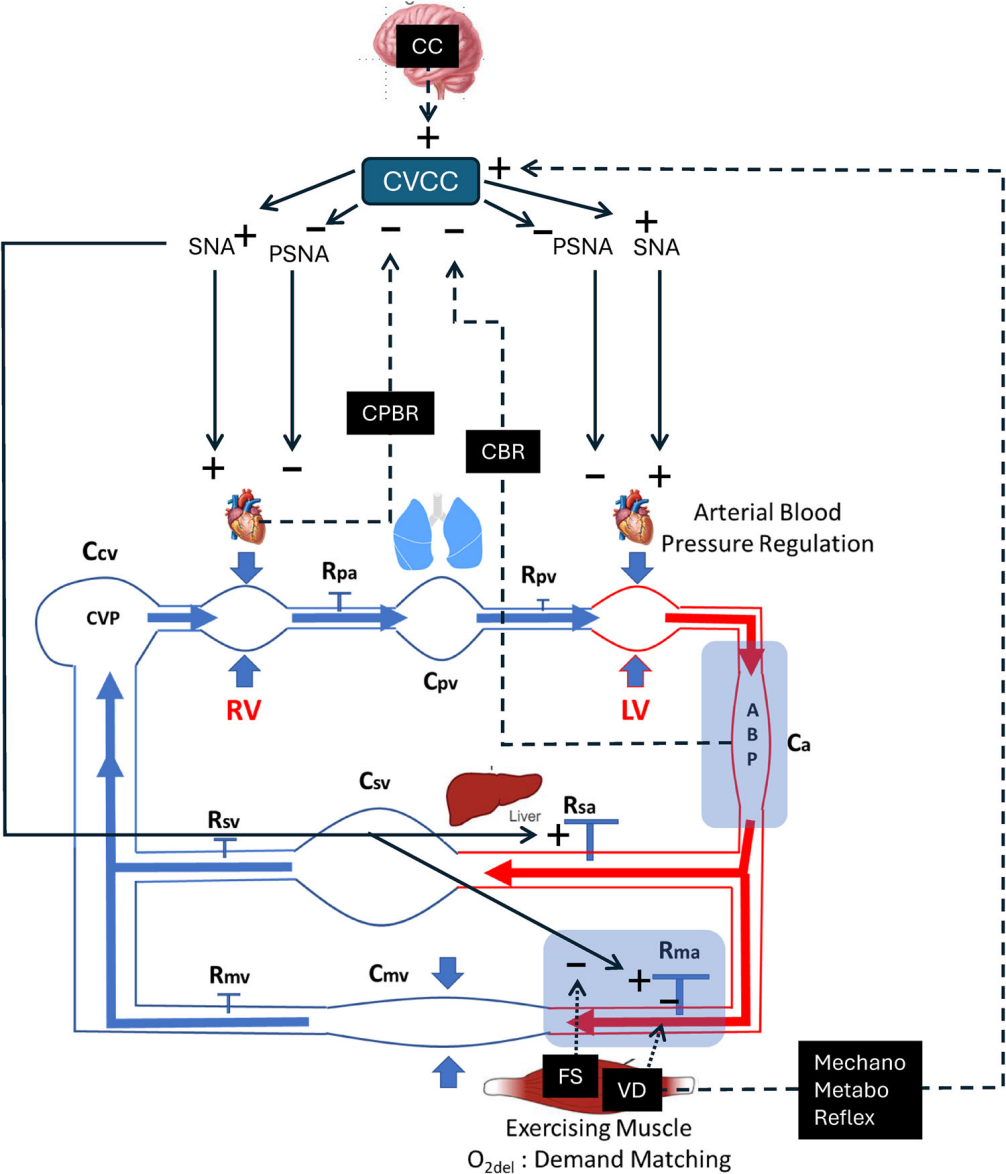
The cardiovascular circuit identifying the site of two key regulated variables shaded in light blue: arterial blood pressure and oxygen delivery–demand matching. Autonomic neural and local vasodilatory mechanisms are activated with exercise. Their interaction determines oxygen delivery–demand matching and arterial blood pressure regulation. Autonomic neural control (sympathetic neural activity ‐ SNA; parasympathetic neural activity ‐ PSNA) is indicated by solid arrows, with ‘+’ and ‘−’ indicating excitatory/activating/increasing effects and inhibitory/deactivating/decreasing effects, respectively. Dashed lines indicate feedback and feedforward influences on the cardiovascular control centre (CVCC), including central command (CC), cardiopulmonary baroreflex (CPBR), carotid baroreflex (CBR), mechano and metaboreflex. Taps represent resistance to blood flow between compartments: muscle arteriole vascular resistance (Rma), splanchnic arteriole vascular resistance (Rsa), muscle venular resistance (Rmv), splanchnic venular resistance (Rsv), pulmonary arteriolar resistance (Rpa) and pulmonary venular resistance (Rpv). Compartments are as follows: arterial compartment (Ca), muscle venous compartment (Cmv), splanchnic venous compartment (Csv), central venous compartment (Ccv) and pulmonary venous compartment (Cpv). Dotted lines represent exercising muscle originating influences on local resistance vessels consisting of direct vasodilator mechanisms (VD) and functional sympatholytic effects of local vasodilators (FS). Inward‐directed blue arrows on compartments represent cardiac muscle contraction of the right (RV) and left (LV) ventricle and exercising skeletal ‘muscle pump’ effects.

The interactions of vasodilatory, sympatholytic and autonomic neural mechanisms in a healthy system drive adjustments in $\dot{Q}$ and PBF and the venous return that links them (altogether, cardiovascular circuit flow) and achieve a target arterial blood pressure (Holwerda et al., 2015; Joyner & Casey, 2015) (Figure 1). The current view on how a balanced $\dot{Q}$ –PBF increase and the resultant steady‐state circuit flow are achieved in exercise primarily favours coordinated autonomic neural effects on the heart (chronotropic and inotropic activation) and resistance vessels (vasoconstriction) in the face of the exercising muscle vasodilatory mechanisms responding to increased metabolic demand (Fadel, 2013; Holwerda et al., 2015; Joyner & Casey, 2015). Specifically, the $\dot{Q}$ increase is determined by cardiac activation, and sympathetic neural vasoconstrictor effects can limit or reduce blood flow in other tissues (Keller et al., 2004, 2003; Rowell, 1991). Furthermore, exercising skeletal muscle blood flow increases in proportion to metabolic demand owing to vasodilatory mechanisms but is restrained by sympathetic adrenergic vasoconstrictor influences (Delp & O'Leary, 2004; O'Leary et al., 1997) (and, potentially, other vasoconstrictors; Hansen et al., 2020; Holwerda et al., 2015) so as not to be in ‘excess’ of the $\dot{Q}$ increase. Sympathetic vasoconstrictor prevention of ‘excess’ vasodilatation specific to exercising muscle is referred to from this point on as ‘sympathetic restraint’. Given that exercising skeletal muscle is the site of most of the available vascular conductance for arterial blood pressure regulation in exercise, sympathetic restraint has emerged as the proposed means by which $\dot{Q}$–PBF balance and targeted arterial blood pressure are achieved (Rowell, 1991, 1997, 2004)

**FIGURE 2:**
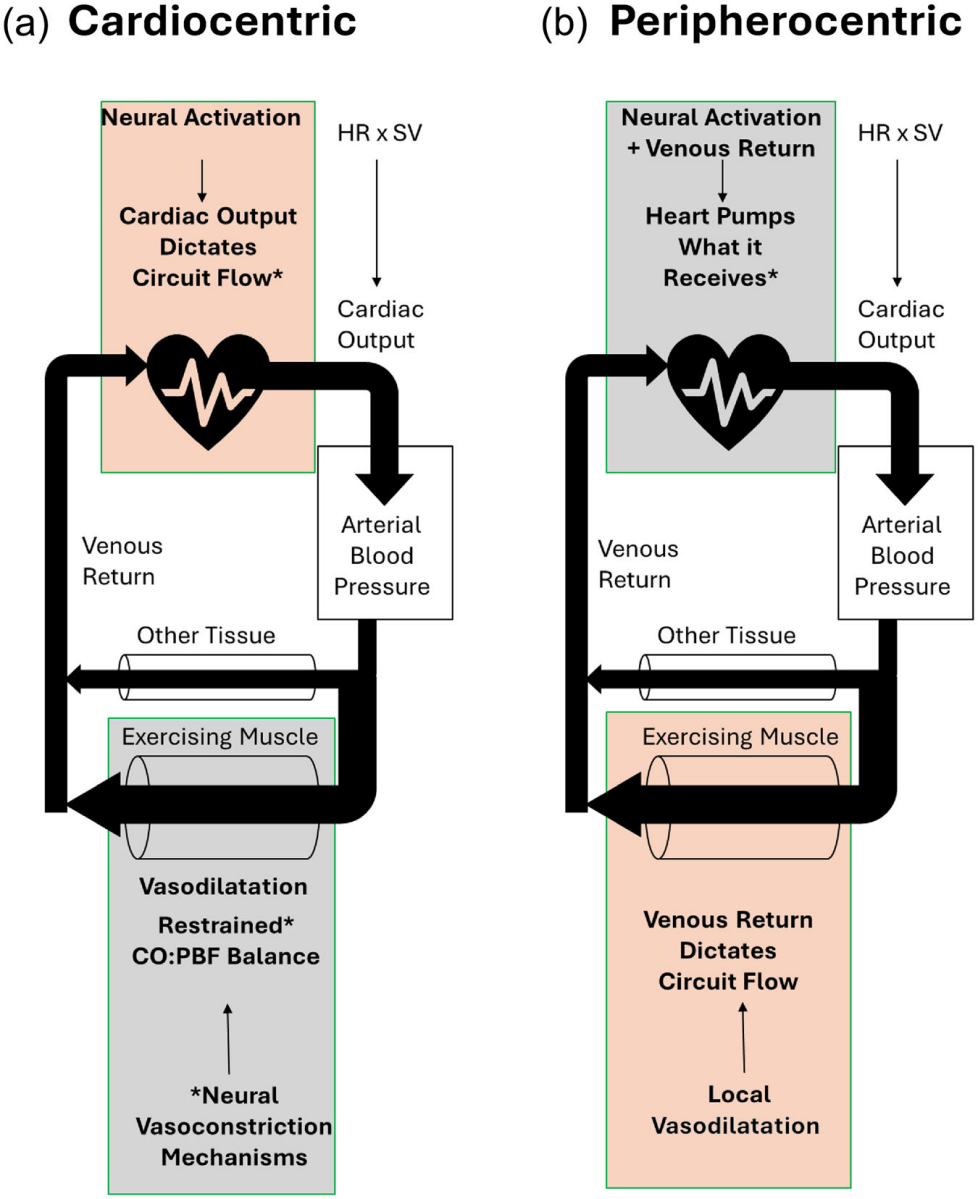
(a) Cardiocentric determination of circuit flow magnitude and balance in submaximal exercise. Activation of the heart determines the rate and magnitude of increase in CO, which is the ‘bottleneck’ for circuit flow increase. Exercising muscle vasodilatation ‘excess’ must be restrained such that CO–PBF balance and target arterial blood pressure are achieved. (b) Peripherocentric determination of circuit flow magnitude and balance in submaximal exercise. Vasodilatation in exercising muscle creates the increase in venous return that allows CO to increase. Autonomic activation of the heart creates a given HR:SV ratio, and the heart pumps whatever venous return it receives. Abbreviations: CO, cardiac output; HR, heart rate; PBF, peripheral blood flow; SV, stroke volume.

This proposed role for sympathetic restraint in submaximal exercise has important implications for cardiovascular control in exercise and the dominance of the heart versus the periphery in determining cardiovascular circuit flow and oxygen delivery–demand matching in exercising muscle. First, it suggests a ‘cardiocentric’ determination of the rate and magnitude of increase in cardiovascular circuit flow in exercise. In other words, the neural activation of the heart in combination with local determinants of cardiac filling dictate the $\dot{Q}$ response and therefore cardiovascular circuit flow for a given exercising muscle metabolic demand. To elaborate, in conditions of a specific increase in $\dot{Q}$, an ‘excess’ vasodilatation in exercising muscle would transiently increase flow out of the arterial compartment, and arterial blood pressure would decrease. But this arterial blood pressure decrease would counteract the increased vasodilatation and quickly bring exercising muscle blood flow back to where it was. Thus, circuit flow balance would be restored at the specific $\dot{Q}$ but with lower arterial blood pressure and higher exercising leg vascular conductance. In other words, if a specific $\dot{Q}$ increase dictates cardiovascular circuit flow, a change in exercising muscle vascular conductance does not alter exercising muscle perfusion or circuit flow. Instead, it determines the arterial blood pressure at that circuit flow. In this way, sympathetic restraint ensures that vasodilatation is not in ‘excess’ of the specific $\dot{Q}$ response, meaning that the target arterial blood pressure is achieved (Figure 2a). Second, given that exercising muscle oxygen delivery is regularly described as being ‘tightly matched’ to metabolic demand in submaximal exercise, it implies that $\dot{Q}$ is closely calibrated to exercising muscle metabolic demand and that the vasodilatory mechanism response, were it to be allowed to express fully, is in excess.

However, considerable evidence exists to call into question the concept that vasodilatation in exercising muscle can be ‘excessive’ relative to a specific $\dot{Q}$ response in submaximal exercise; instead, vasodilatation dictates the cardiovascular circuit flow for a given exercise intensity (Bada et al., 2012; Gonzalez‐Alonso et al., 2008; Radegran & Calbet, 2001; Rosenmeier et al., 2004), i.e., a ‘peripherocentric’ determination of cardiovascular circuit flow (Figure 2b). We therefore propose that the present view of sympathetic restraint requires scrutiny and that cardiocentric versus peripherocentric implications for cardiovascular circuit flow in exercise require further investigation.

This brief review examines the phenomenon of sympathetic restraint critically, in part from a teleological perspective, in the context of cardiac–peripheral response balance in exercise, where exercising muscle vasodilatation is the disturbance challenging $\dot{Q}$–PBF balance and protection of arterial blood pressure. We confine ourselves to sympathetic vasoconstriction, acknowledging that non‐adrenergic vasoconstriction in exercising muscle has also been identified (Barrett‐O'Keefe et al., 2013; Hansen et al., 2020; Wray et al., 2007). Recognizing that ageing (Hearon & Dinenno, 2016; Poole‐Wilson et al., 1992; Sullivan et al., 1989) and disease can compromise cardiac function and increase the magnitude of sympathetic restraint, we also confine ourselves to a young, healthy system. We hope this will stimulate: (1) research efforts to establish the role(s) of exercising muscle sympathetic restraint in submaximal exercise definitively; and (2) advance insight into cardiac–peripheral interactions in the creation of cardiovascular circuit flow and effective $\dot{Q}$–PBF balance and arterial pressurization in the face of substantial exercising muscle vasodilatation in exercise.

## 2 EVIDENCE FOR SYMPATHETIC RESTRAINT IN SUBMAXIMALLY EXERCISING MUSCLE

Exercising muscle does experience an increase in muscle sympathetic nerve activity with exercise (Boulton et al., 2018). Experimental interventions that elevate sympathetic neural activation or stimulation of α‐adrenergic receptors demonstrate vasoconstriction in exercising limbs. For example, infusion of α‐receptor agonists or tyramine, which evokes noradrenaline release from sympathetic nerve terminals in exercising limbs, evokes exercising limb vasoconstriction (Heinonen et al., 2013; Rosenmeier et al., 2003; Tschakovsky et al., 2002). Positive neck pressure to evoke baroreflex‐mediated increases in sympathetic outflow causes vasoconstriction in exercising limbs (Keller et al., 2004, 2003). Furthermore, addition of exercising skeletal muscle mass (Calbet et al., 2004; Secher et al., 1977; Volianitis et al., 2003) and increased respiratory muscle work (Katayama et al., 2019; Sheel et al., 2018) will cause vasoconstriction in already exercising limbs when combined muscle mass is large. Interestingly, exercising limb vasoconstriction also happens when the perfusion requirements of combined exercising muscle mass are well below the pumping capacity of the heart (Hansen et al., 2020; Kagaya et al., 1994; Saito et al., 1992), indicating that such sympathetic restraint is not limited to preventing exercising muscle vasodilatation from exceeding cardiac pumping capacity. Of note, sympathetic vasoconstrictor influences are blunted in exercising muscle in what appears to be an exercise intensity‐dependent manner, a phenomenon termed functional sympatholysis (Dinenno & Joyner, 2003; Ruble et al., 2002; Tschakovsky et al., 2002). However, this does not prevent addition of sympathetic vasoconstrictor influence from being able to affect exercising limb vascular conductance (Keller et al., 2004, 2003).

Although the aforementioned approaches demonstrate that exercising limb vasculature (typically assumed to be exercising muscle resistance vessels) is susceptible to increased sympathetic adrenergic vasoconstriction, they do not confirm that the normally existing state of vasodilatation is sympathetically restrained. Evidence for tonic vasoconstriction as a determinant of the existing resistance vessel tone in exercising muscle stems from experiments in which sympathetic adrenergic vasoconstriction is blocked or reduced. In treadmill‐exercising dogs, non‐selective and selective blockade of exercising hindlimb α‐receptors results in increased exercising limb vascular conductance (Buckwalter & Clifford, 1999; Buckwalter et al., 1997; O'Leary et al., 1997; Stickland et al., 2009). In humans, Joyner et al. (1992) performed stellate ganglion blockade to eliminate sympathetic outflow to the arm and found that the increase in exercising forearm blood flow was greater compared with control. Hansen et al. (2022, 2020) observed similar effects with α‐receptor blockade during hand‐grip exercise. The current consensus is that these limb blockade studies reveal a degree of sympathetic vasoconstriction in the exercising muscles of the limb in normal conditions. However, in the only study that used imaging techniques to assess exercising and non‐exercising muscle within an exercising limb, Heinonen et al. (2013) found that infusion of an α‐receptor antagonist failed to alter exercising quadriceps muscle blood flow, while increasing non‐exercising hamstring muscle blood flow. These findings support the existence of sympathetic restraint in non‐exercising muscle in an exercising limb and were interpreted to indicate that sympathetic restraint is important for between‐muscle blood flow distribution. However, the lack of effect of an α‐receptor antagonist on perfusion of exercising muscle calls into question whether exercising muscle sympathetic restraint is necessarily in operation (Tschakovsky, 2014) and whether the findings of limb α‐receptor blocker infusion study require a more nuanced interpretation.

In summary, although functional sympatholysis blunts sympathetic vasoconstriction in exercising muscle (Buckwalter & Clifford, 1998; Rosenmeier et al., 2008), sympathetic adrenergic activity increases in exercising limbs (Boulton et al., 2018; Savard et al., 1987) and can restrain vasodilatation (Buckwalter & Clifford, 1999; Buckwalter et al., 1997; Hansen et al., 2022, 2020), and such restraint can be used by the arterial baroreflex to protect arterial blood pressure if hypotension is sensed (Keller et al., 2004, 2003). Although the existence of sympathetic restraint is well supported, we believe the current consensus that this sympathetic restraint is necessary to prevent ‘excess’ exercising muscle vasodilatation from exceeding the ‘normal’ $\dot{Q}$ response to exercise (Holwerda et al., 2015; Joyner & Casey, 2015) requires scrutiny. What is the experimental evidence that has led to this consensus, and what is the evidence suggesting that the consensus requires scrutiny?

## 3 THE NECESSITY OF SYMPATHETIC RESTRAINT FOR PREVENTING ‘EXCESS’ VASODILATATION IN SUBMAXIMAL EXERCISE: WEIGHING THE EVIDENCE

The discovery that skeletal muscle has a vasodilatory capacity to allow 200–300 ml/100 g/min of blood flow in the classic studies in the 1980s by Andersen and Saltin (1985) and Rowell et al. (1986) revealed that the cardiac pumping capacity can be exceeded by exercising muscle vasodilatory capacity during large muscle mass exercise. This led Rowell (1991, 1997) to propose that sympathetic vasoconstrictor restraint of exercising muscle vasodilatation was necessary to balance $\dot{Q}$ and peripheral vasodilatation near maximal $\dot{Q}$ capacity. However, in submaximal exercise, cardiac pumping capacity is not threatened, yet the current interpretation of sympathetic restraint even in submaximal exercise is that arterial blood pressure would be compromised without it preventing ‘excess’ exercising muscle vasodilatation (Holwerda et al., 2015; Joyner & Casey, 2015; Rowell, 1997, 2004).

### 3.1 Evidence from multi‐limb exercise models

Studies of large muscle mass exercise, in which arm exercise is added to leg exercise or vice versa, demonstrate that the maximal vasodilatation observed in isolated upper or lower limb exercise would combine to be greater than that observed in combined upper and lower limb exercise (Calbet et al., 2004; Secher et al., 1977; Volianitis et al., 2003). As such, a reduction in exercising limb vascular conductance is observed with combined upper and lower limb exercise and interpreted as sympathetic restraint necessary to protect arterial blood pressure. Certainly, the size of muscle mass involved and the exercise intensities in these studies resulted in the potential for cardiac pumping capacity to be exceeded by exercising muscle vasodilatation. Consequently, protection of arterial blood pressure required sympathetic restraint.

But what of the findings that vasoconstrictor restraint of exercising muscle initiated with recruitment of additional exercising muscle where cardiac pumping capacity is not threatened (Hansen et al., 2020; Saito et al., 1992)? The recent study by Hansen et al. (2020) is particularly interesting in this regard. When moderate leg cycling exercise was added to rhythmic submaximal forearm hand‐grip exercise, exercising forearm blood flow decreased owing to vasoconstriction. The vasoconstriction was not limited to adrenergic mechanisms, hence, as mentioned previously, exercising muscle blood flow restraint is not only achieved by sympathetic activity. What is important to consider here is that there was no need to reduce forearm blood flow to ensure that exercising muscle blood flow requirements could be met without exceeding the $\dot{Q}$. This suggests that sympathetic restraint of exercising muscle perfusion by additional exercising muscle is an occurrence that is not necessarily about being ‘necessary’ for preventing ‘excess’ vasodilatation and thereby imbalance of $\dot{Q}$–PBF leading to reduced arterial blood pressure.

### 3.2 Evidence from autonomic failure and systemic adrenergic blockade

The commonly cited evidence in humans for sympathetic restraint of ‘excess’ vasodilatation is the reduction rather than normal increase in arterial blood pressure, even in mild exercise, experienced by patients with autonomic failure, spinal cord injury patients or those having surgical lumbar sympathectomy (Akinola et al., 1999; Marshall et al., 1961; Puvi‐Rajasingham et al., 1997; Smith & Mathias, 1996; Smith et al., 1996, 1995). Hypotension with exercise in autonomic failure is attributed to the loss of vasoconstriction specific to exercising muscle (Calbet & Joyner, 2010; Joyner & Casey, 2015; Rowell, 1991, 1997). But there are problems with interpreting findings in this disease model as being relevant for a healthy system.

**FIGURE 3:**
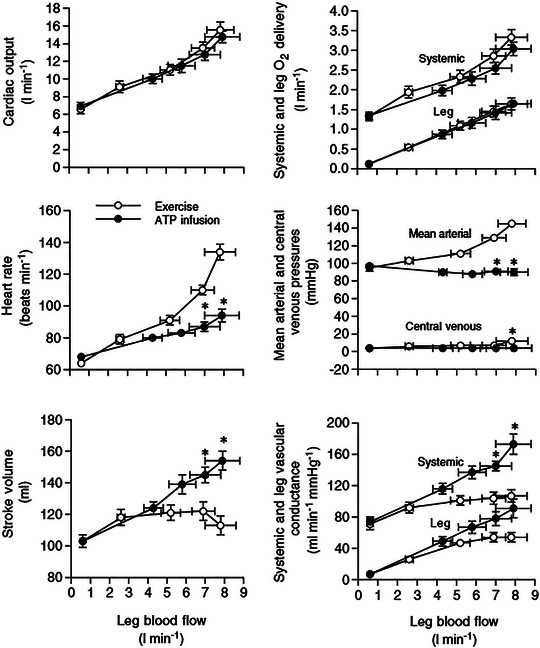
Comparison of femoral artery vasodilator (ATP) infusion versus knee‐extension exercise. Leg blood flow increases on the x‐axis with increasing exercise intensity and vasodilator infusion. With dilator infusion, a much lower heart rate response occurred, and stroke volume continued to increase well above the limit observed in exercise. Continued proportional increases in leg blood flow were driven by vasodilatation. In exercise, a pressor response and noticeable restraint of exercising leg vasodilatation combined to increase leg blood flow at higher exercise intensities. *Significantly different from exercise at the same time point. Reproduced from Gonzalez‐Alonso et al. (2008) with permission.

First, patients with autonomic failure lack the capacity to increase sympathetically mediated vasoconstriction and venoconstriction throughout the cardiovascular circuit. This is an important distinction from healthy humans, because autonomic failure patients would be unable to reduce inflow to the compliant peripheral venous beds (dependent on the balance between vasoconstriction and vasodilatation) and decrease the contained volume (dependent on venoconstriction) in all regions and, in particular, the splanchnic region, which is the region with the largest capacitance in the human body that is normally constricted during exercise (Bergeron et al., 2001; Chaudhuri et al., 1992). Second, and perhaps even more important, is the commonly observed blunted heart rate response to exercise of ≤50% (Akinola et al., 1999; Puvi‐Rajasingham et al., 1997; Smith & Mathias, 1996). ‘Excess’ vasodilatation should increase venous return in patients with autonomic failure, but if the heart rate response is limiting the increase in $\dot{Q}$, a mismatch between $\dot{Q}$ and peripheral blood flow is created by loss of ability for the heart to respond appropriately. In other words, if in a healthy system, $\dot{Q}$ –PBF balance was a function of exercising muscle vasodilatation driving venous return and the heart responding by transferring what it receives to the arterial circulation (Gonzalez‐Alonso et al., 2008); see Figure 3], hypotension in autonomic failure would be a result of a cardiac response deficiency that would not be relevant for the healthy system.

In healthy persons, systemic infusion of α‐adrenergic receptor antagonists can be used to compromise restraint of vascular conductance at the systemic level. Accordingly, McLeod et al. (1984) systemically infused prazosin (selective α_1_‐adrenergic receptor antagonist) during exercise and compared the blood pressure response against a within‐participant placebo. During cycling exercise (50–200 W) with prazosin infusion, mean arterial blood pressure was reduced by an average of 8 mmHg (∼9%) compared with placebo at the same workload. Similar to the work of McLeod et al. (1984), Sugawara et al. (2013) had participants complete single‐leg‐extension exercise with and without systemic prazosin infusion. Mean arterial blood pressure did not change significantly at rest. However, during exercise the mean arterial blood pressure decreased by 9 mmHg during rhythmic 40% and 60% maximal voluntary extensor contraction force exercise with prazosin infusion. Therefore, in healthy persons, systemic selective α_1_‐adrenergic receptor antagonist infusion compromises mean arterial blood pressure. Of note, in this experimental model, α_2_‐adrenergic receptors would be unaffected. Therefore, unlike patients with autonomic failure, these healthy persons would have some capacity for sympathetic vasoconstriction during exercise in response to a decrease in arterial blood pressure. Nevertheless, like the work in patients with autonomic failure, it is evident that systemic sympathetic vasoconstrictor capability is essential in healthy humans for achieving the normal blood pressure response. Nonetheless, this does not mean that sympathetic restraint specific to exercising muscle is necessary; instead, it suggests that systemic autonomic control of resistance vessels is necessary.

In summary, compromise to systemic autonomic control results in hypotension during submaximal exercise. The hypotension is commonly attributed to lack of restraint of vasodilatation of exercising muscle, without considering issues with the cardiac response capability in persons with autonomic failure (Calbet & Joyner, 2010; Joyner & Casey, 2015).

### 3.3 Evidence from exercising limb adrenergic blockade

The ideal approach to determine the role of sympathetic restraint in submaximal exercise would be adrenergic blockade specific to the exercising muscle. Adrenergic blockade has been performed in the exercising forearm either in isolation (Joyner et al., 1992) or combined with moderate leg exercise (Hansen et al., 2020) and demonstrated increased forearm vascular conductance indicative of existing sympathetic restraint. However, the forearm blood flow changes are essentially insignificant in the context of challenging $\dot{Q}$–PBF balance. A leg exercise model is required, in which substantive vasodilatation and $\dot{Q}$ increase can be achieved in submaximal exercise.

Such a model has been used in treadmill‐exercising dogs. Buckwalter et al. (1997, 1999) performed selective adrenergic receptor blocker bolus infusion into the arterial supply of one hindlimb during exercise at 3, 6 and 6 mph + 10% treadmill grade. Infusion caused an increase in experimental hindlimb vascular conductance and blood flow, with no change in the contralateral hindlimb. These findings demonstrate sympathetic restraint in the exercising limbs of dogs. Importantly, there was no decline in arterial blood pressure with the ‘excess’ hindlimb vasodilatation. This means either that $\dot{Q}$ increased to balance the increased hindlimb blood flow and/or that other vascular beds were constricted. Which of these responses occurred could not be determined, because only heart rate was reported (and although it did not change, stroke volume could have changed). These findings argue against the necessity for sympathetic restraint of an exercising limb to maintain arterial blood pressure.

In contrast, the same treadmill‐exercising dog model was used by O'Leary et al. (1997), but with adrenergic blocker bolus infusion to both hindlimbs during exercise at 3.2, 6.4, 6.4 km/h + 10% grade, and 8 km/h + 15% grade. Terminal aortic flow to the hindlimbs increased with increased hindlimb vasodilatation at all exercise intensities, indicating underlying sympathetic restraint of the hindlimbs. These investigators did observe an increase in heart rate with adrenergic blocker infusion, and a decline in arterial blood pressure ranging from ∼3 to 9 mmHg depending on work rate. Stickland et al. (2009) also performed dual hindlimb adrenergic blocker infusion in dogs exercising on a treadmill, but with repeated infusions to maintain blockade efficacy over the course of exercise. The infusion increased hindlimb vascular conductance at both the 2.5 mph 5% grade and 4 mph 10% grade intensities, consistent with existing sympathetic restraint. Furthermore, there were marked increases in heart rate of 57–70 beats/min and marked declines in arterial blood pressure of ∼18 mmHg. These two experiments support the contention that a loss of sympathetic restraint of ‘excess’ vasodilatation in exercising limbs leads to hypotension in dogs. The lack of such an effect in the studies by Buckwalter et al. (1997, 1999) might be attributable to the magnitude of exercising limb sympathetic restraint that was removed (one hindlimb vs. two hindlimbs).

In humans, we are aware of two studies in which lower limb adrenergic blockade was performed during knee‐extension exercise. Addressing the most recent first, Barrett O'Keefe et al. (2019) performed common femoral artery Doppler ultrasound measurements and observed increased exercising leg vascular conductance and blood flow with adrenergic blockade and an ∼5% decline in arterial blood pressure. Note that this was limb vascular conductance and blood flow, and only some of the limb muscle mass (quadriceps) was exercising. In contrast, Heinonen et al. (2013) used positron‐emission tomography scanning to quantify blood flow responses to specific upper leg muscles during knee‐extension exercise. Femoral artery infusion of the non‐selective α‐receptor blocker phentolamine did not increase exercising quadriceps muscle blood flow or its perfusion heterogeneity. What did occur was increased resting hamstring muscle blood flow. Thus, total limb perfusion increased via an ‘excess’ vasodilatation of non‐exercising muscle, not exercising muscle. The mean value for arterial blood pressure during exercise was 6 mmHg lower in blockade conditions, but this was not statistically significant. However, this might be a case of inadequate power, with nine participants and a variability of more than twice the difference between means. Nonetheless, heart rate was significantly elevated in blockade. If the increase in heart rate observed with blockade reflected a $\dot{Q}$ increase, then the findings of Heinonen et al. (2013) are consistent with an increased limb perfusion being balanced by the response of the heart to maintain $\dot{Q}$–PBF balance and arterial blood pressure. This study is particularly important because it identified inactive muscles as the site of existing sympathetic restraint in an exercising limb but found no such restraint in the exercising quadriceps muscles. This has important implications for the total leg blood flow and vascular conductance increases and the mild blood pressure compromise observed by Barrett O'Keefe et al. (2019).

In summary, adrenergic blockade in exercising limbs when performed in dogs suggests that excess vasodilatation in one exercising hindlimb does not affect arterial blood pressure, but if occurring in both hindlimbs, hypotension results. Furthermore, despite a substantial increase in heart rate, the increase in $\dot{Q}$ does not protect arterial blood pressure. Keeping in mind the potential for species differences, in humans the necessary studies for a definitive testing of whether ‘excess’ vasodilatation created by removal of exercising muscle sympathetic restraint compromises $\dot{Q}$–PBF balance and thereby arterial blood pressure are lacking. However, as we shall see in the next section, ‘excess’ vasodilatation can be created via vasodilator infusion, and this will call into question the current conceptualization of $\dot{Q}$–PBF balance being threatened if sympathetic restraint of ‘excess’ vasodilatation does not occur.

### 3.4 Evidence from limb vasodilator infusion

At rest there is a $\dot{Q}$–PBF balance, with a given level of lower limb vascular conductance. In this scenario, femoral artery infusion of vasodilators essentially creates an ‘excess’ vasodilatation in the lower limb. There are no exercise‐generated cardiac activation signals, such as central command, muscle metaboreflex or mechanoreflex, with this experimental intervention. There could be cardiopulmonary baroreflex (changes in central blood volume) or carotid and aortic baroreflex (peripheral vasodilatation decreasing arterial blood pressure)‐mediated alterations in autonomic neural activity to the heart and resistance vessels. Suffice it to say that this is a situation where cardiac activation is reactionary, rather than initiating. The same is the case in a submaximally exercising lower limb; infusion of vasodilators would increase vasodilatation above the exercise target vasodilatation, i.e., create ‘excess’ vasodilatation. However, this would be on a background of exercise signals mediating autonomic activation. What is observed?

Radegran and Calbet (2001) created ‘excess’ vasodilatation via femoral artery infusion of adenosine during steady‐state one‐leg dynamic knee‐extension exercise at ∼63% of maximal work rate. The highest dose of adenosine increased exercising leg blood flow from ∼5 to almost 8 L/min. In response to vasodilator infusion, heart rate increased from ∼110 to ∼130 beats/min. Arterial blood pressure was not compromised. Rosenmeier et al. (2004) performed femoral artery infusion of the vasodilators adenosine and ATP at rest to increase resting leg blood flow to ∼ 3 L/min, similar to what was observed in mild knee‐extension exercise. No differences in arterial blood pressure were observed. Furthermore, addition of combined adenosine and femoral artery infusion of ATP during knee‐extension exercise increased leg blood flow from 3 L/min at exercise steady state to 7 L/min, i.e., 4 L/min in ‘excess’ of the normal exercising limb perfusion response. Again, this ‘excess’ vasodilatation did not compromise arterial blood pressure. Heart rate increased from ∼90 to ∼110 beats/min with vasodilator infusion. There was no increase in femoral venous noradrenaline concentration, suggesting no peripheral sympathetic restraint to manage arterial blood pressure. However, it must be acknowledged that limb noradrenaline spillover into venous effluent might not provide adequate sensitivity for detecting increased noradrenaline release from sympathetic nerve terminals.

Gonzalez‐Alonso et al. (2008) added measures of $\dot{Q}$ to their comparisons of progressive knee‐extension exercise with resting leg infusion of ATP at increasing doses to match exercising limb blood flow responses (see Figure 3). What emerged from this study was a striking difference in the way increased $\dot{Q}$ was achieved in response to resting limb vasodilatation versus exercise. With exercise, there was elevated stroke volume with the initial few increases in exercise intensity, along with heart rate increases. However, further increases in exercise intensity resulted in only increases in heart rate, as $\dot{Q}$ increased in proportion to exercising leg blood flow. In contrast, with vasodilator infusion in a resting leg, the stroke volume continued to increase with increasing vasodilator dose, with a much lower heart rate response. A further striking difference was that at the higher end of the limb blood flows achieved, the continued proportional increase in leg blood flow and $\dot{Q}$ was driven by a pressor response in exercise with vasodilatation restrained, while continued increasing vasodilatation in the resting limb infusion with no change in arterial blood pressure drove the increase in flow. The difference in blood pressure between exercise and resting limb vasodilator infusion does not represent hypotension in the infusion condition, because the target blood pressure would remain the resting blood pressure. In both conditions, the $\dot{Q}$ increased in proportion to leg blood flow.

Bada et al. (2012) added atrial pacing once steady‐state exercising or resting vasodilator infusion‐mediated increases in leg blood flow and $\dot{Q}$ had been reached. Again, vasodilator infusion into a resting limb to create the same increase in perfusion as exercise did not compromise arterial blood pressure. Furthermore, increasing heart rate via atrial pacing did not augment $\dot{Q}$ in exercise or during resting limb vasodilator infusion. The interpretation was that at higher circuit flow rates, the heart cannot elevate circuit flow independently; instead, it is venous return that determines $\dot{Q}$.

Collectively, limb vasodilator infusion studies consistently demonstrate that ‘excess’ vasodilatation relative to the steady‐state oxygen delivery–demand matching at rest or during exercise does not compromise arterial blood pressure in healthy individuals. Instead, the heart responds to increased venous return and ‘pumps what it receives’ such that $\dot{Q}$–PBF balance and arterial blood pressure are maintained. This occurs without the normal exercise‐evoked autonomic neural activation mechanisms, such as central command or exercising muscle mechanoreflex and metaboreflex feedback. It is consistent with the cardiovascular circuit flow being dictated by peripheral vasodilatation and the heart pumping what is returned to it, as illustrated in the depiction in Figure 2b of a peripherocentric determination of cardiovascular circuit flow. There is, however, a limitation to the reported findings of these studies, in that they reflect the system once it has stabilized. There is no reporting of the dynamic response to the initiation of ‘excess’ vasodilatation. Therefore, we do not know whether the onset of ‘excess’ limb vasodilatation without simultaneous exercise‐induced autonomic activation creates a $\dot{Q}$–PBF imbalance and transient hypotension, such that it is a baroreflex‐mediated adjustment of other tissue vascular conductance that is required to reach the steady‐state $\dot{Q}$–PBF balance and normalized arterial blood pressure. A transient hypotension occurs in the response to a significant, sudden reactive hyperaemia induced by release of short periods of lower limb occlusion (Ogoh et al., 2010), triggering a baroreflex‐mediated response. Often, a transient decline followed by rapid recovery of arterial blood pressure is also observed at the onset of moderate supine leg cycling (Toska & Eriksen, 1994) and upright cycling (Wieling et al., 1996). Nevertheless, vasodilator infusion studies clearly demonstrate that a $\dot{Q}$–PBF balance with appropriate arterial blood pressure can be reached when ‘excess’ vasodilatation is created.

On a side note, but with additional potentially important ramifications for understanding the integrated cardiovascular response to exercise, a particularly intriguing observation of the response by the heart to limb vasodilator infusion is the observation that stroke volume could increase well above what is observed in exercise for the same $\dot{Q}$. This stroke volume response argues against the current view that increases in heart rate are required for increasing $\dot{Q}$ beyond where the plateau in stroke volume occurred in exercise. It begs the question, if the observed heart rate response during exercise was not necessary for achievement of the required $\dot{Q}$, why does it occur? Is there something important about ‘partitioning’ the venous return such that those partitions do not exceed the maximal observed stroke volume? One possibility in this regard is that an important purpose of increasing heart rate is to prevent what, for a given heart, would represent ‘overfilling’ and potentially excess pulse pressure in the pulmonary circulation. In other words, does it have a protective role? To our knowledge, consideration of the heart rate relative to stroke volume response in exercise has not been considered in terms of protecting either ventricular filling stress on the heart or effects of pulse pressure on the fragile pulmonary microcirculation.

### 3.5 Summary

Examination of the current body of evidence identifies the problems with interpreting responses of autonomic failure patients to submaximal exercise in the context of sympathetic restraint necessity for prevention of ‘excess’ exercising muscle vasodilatation. Most compelling in this regard are the vasodilator infusion studies that create ‘excess’ vasodilatation yet do not result in any compromise to arterial blood pressure. However, observations in dogs do suggest that, for that species, removal of sympathetic restraint in exercising limbs can create considerable hypotension. The necessary exercising limb adrenergic blockade studies are currently absent in humans. The findings by Heinonen et al. (2013) suggest that exercise models in which all muscles are engaged are required for lower limb adrenergic blockade studies to interpret responses as stemming from removal of exercising muscle sympathetic restraint. Certainly, the observations in the literature require that we scrutinize the current view that sympathetic vasoconstrictor restraint of ‘excess’ vasodilatation in exercising muscle is necessary to prevent peripheral blood flow from exceeding the cardiac response to exercise at intensities where $\dot{Q}$ capacity is not limited. Furthermore, we need to consider what that view and the contradicting evidence imply about how cardiovascular circuit flow balance is achieved during exercise.

## 4 IMPLICATIONS OF THE CURRENTLY ASSUMED ROLE OF SYMPATHETIC RESTRAINT FOR THE UNDERLYING NATURE OF INTEGRATED CARDIOVASCULAR CONTROL IN EXERCISE

The ‘disturbance’ of exercising muscle to the existing steady‐state circuit flow balance at any level of activity is the increased energy demand for force production. Support for continued force production requires provision of energy via aerobic metabolism and the dissipation of heat and metabolic byproducts. For both functions, increased perfusion of exercising muscle must occur at levels that are at least adequate (Nyberg & Jones, 2022), if not optimal. Indeed, a rapid and proportional increase in blood flow with exercise intensity is interpreted as a ‘tight matching’ of perfusion with metabolic demand (Boushel, 2003; Clifford & Hellsten, 2004). Perfusion of exercising muscle is a function of the pressure gradient and the conductance for flow. The former depends on an adequate arterial blood pressure and the latter is determined by resistance vessel tone. Initiation of considerable vasodilatation by local vasodilatory mechanisms that are activated in proportion to muscle excitation and increased metabolism is essential for increased perfusion. However, in larger muscle mass exercise, such vasodilatation in and of itself would quickly lead to hypotension (i.e. reductions in the pressure gradient for flow) if a co‐ordinated increase in $\dot{Q}$ did not occur. Thus, in the face of elevated perfusion requirements, arterial blood pressure regulation is challenged.

Revisiting Figure 1, we know that the heart and blood vessels are controlled through autonomic neural, hormonal and local vasoregulatory mechanisms that are evident in the response to exercise. Elevated sympathetic adrenergic vasoconstrictor activity to resistance vessels is a well‐established part of the cardiovascular control response to exercise (DeLorey, 2021; Fadel et al., 2001; Saito et al., 1993; Victor et al., 1987), and the activity directed at exercising skeletal muscle is currently believed to be necessary to achieve the required balance of peripheral vasodilatation with the $\dot{Q}$ response to exercise by preventing ‘excess’ exercising muscle vasodilatation (Joyner & Casey, 2015). Two key implications of such a role for cardiovascular control in exercise require consideration.

### 4.1 Currently proposed role of sympathetic restraint implies a targeted $\dot{Q}$ response to exercising muscle requirements for perfusion

The notion of ‘excess’ vasodilatation is clearly relevant for exercise intensities at which the required cardiovascular circuit flow approaches $\dot{Q}$ capacity, because of the vasodilatory capacity of the ‘sleeping giant’ that is skeletal muscle (Andersen & Saltin, 1985; Richardson et al., 2000). But in submaximal exercise, a pumping capacity limit on $\dot{Q}$ is not in question, because further increases in exercise intensity are met with increased exercising muscle vasodilatation and $\dot{Q}$ (Mortensen et al., 2005). The currently proposed role of ‘excess’ vasodilatation prevention requires accepting a set response of cardiac activation for a given intensity of submaximal exercise as the determinant of the total circuit flow response in exercise (see Figure 2a). Furthermore, it requires that vasodilatory signals in exercising muscle are not ensuring that perfusion matching to demand is met, but instead are in excess, and it is the activation of the heart that is calibrated to perfusion demands of the muscle, requiring restraint of vasodilatory effects.

There is evidence supporting cardiac activation as the bottleneck for the increase in cardiovascular circuit flow in submaximal exercise. Systemic administration of cardiac β‐receptor blockade slows the rate of adjustment in $\dot{Q}$ (Hughson & Kowalchuk, 1991; Kowalchuk et al., 1989) and can reduce the steady‐state $\dot{Q}$ (Pawelczyk et al., 1992). Heart rate adjustment is slower for transitions from prior exercise to higher exercise intensity versus rest to exercise (exercise to exercise relies on slower sympathetic cardiac activation) and is accompanied by a slower rate of increase in oxygen uptake (Hughson & Morrissey, 1982, 1983). Amann et al. (2011) have observed a reduction in $\dot{Q}$ during knee‐extensor exercise when type III/IV muscle afferents were blocked. Their additional observation of a reduced arterial blood pressure and exercising leg blood flow, along with apparent increases in exercising limb noradrenaline spillover, are certainly consistent with reductions in cardiac activation triggering sympathetic restraint in an exercising limb to mitigate compromise to arterial blood pressure. Collectively, these studies demonstrate that if the neural activation of the heart is compromised, then its reduced response determines the cardiovascular circuit flow in exercise. The findings by Amann et al. (2011) would indicate that autonomic activation of the heart triggered by sensing of muscle activity is important in creating the expected cardiac output response to exercise. However, all the above‐mentioned studies have a limitation for understanding an intact system. If it is peripheral vasodilatation that determines venous return, as can occur with vasodilator infusion both at rest and during exercise (Bada et al., 2012; Gonzalez‐Alonso et al., 2008; Radegran & Calbet, 2001; Rosenmeier et al., 2004), and the heart can respond to this by pumping what it receives if it has normal capacity for activation, then compromising its activation cannot determine whether the normal circuit flow is determined cardiocentrically.

None of this is to say that sympathetic restraint is not occurring, nor that it does not have an important role in the integrated control of cardiovascular responses to exercise. Physiological control systems are characterized by simultaneous competing influences, the balance of which results in the observed response. The well‐established ability of the baroreflex to alter vascular conductance of exercising muscle by adjusting sympathetic vasoconstriction (Keller et al., 2004, 2003; Wray et al., 2004) speaks to the existence and need for such control to ensure arterial blood pressure protection when it is disturbed. But this is a different context from being necessary to prevent what would be an ‘excess’ vasodilatation response that exceeds a set cardiac output response to a given submaximal exercise intensity.

We propose that the existence of sympathetic vasoconstrictor influence in exercising muscle might reflect a ‘one foot on the gas, one foot on the brake’ nature of cardiovascular control, which enables responses to deal with arterial blood pressure regulation challenges, should they arise. However, the findings of vasodilator infusion studies (Bada et al., 2012; Gonzalez‐Alonso et al., 2008; Radegran & Calbet, 2001; Rosenmeier et al., 2004) seriously call into question the notion of controlling ‘excess’ vasodilatation relative to a set cardiac output response for a given exercise intensity.

### 4.2 Current proposed role of sympathetic restraint implies that skeletal muscle perfusion requirements are communicated to the heart

The view of sympathetic restraint as necessary to prevent excess exercising muscle vasodilatation has also been advanced for small muscle mass exercise. Specifically, Hansen et al. (2022) concluded that greater adrenergic restraint of exercising forearm muscle blood flow after acclimatization to altitude was ‘necessary to match oxygen delivery to demand and prevent over perfusion of contracting muscle at high altitude’. This view places sympathetic restraint in the position of oxygen delivery–demand matching, whereby vasodilator responses are inherently in excess and require blunting by sympathetic vasoconstrictor influence. Although the forearm exercise model in the paper by Hansen et al. (2022) would not require $\dot{Q}$ adjustments, the construct that the role of adrenergic vasoconstrictor activity in exercising muscle is to prevent overperfusion again implies that ‘excess’ vasodilatory influences must be blunted. In larger muscle mass exercise, when increases in $\dot{Q}$ as part of $\dot{Q}$–PBF balance are necessary, this would imply that the $\dot{Q}$ response is calibrated correctly to muscle metabolic demand and that sympathetic restraint represents the control mechanism for ensuring vasodilatation is constrained accordingly.

In the context of both blood pressure regulation and excess oxygen delivery, the concept of ‘excess’ muscle vasodilatation also implies that normal exercising muscle perfusion is optimal for supporting contracting muscle and that there is no benefit of increased oxygen delivery. It is true that oxygen uptake in submaximal exercise is not improved with increased oxygen delivery above normal, indicating that delivery is adequate to support a given rate of oxidative phosphorylation (Nyberg & Jones, 2022). But this ignores the beneficial effects on muscle metabolic and contractile performance that occur with improving oxygen delivery above normal (Drouin et al., 2022, 2019, 2023; Haseler et al., 1998; Hogan et al., 1992), which is the basis for blood doping improvements in submaximal exercise performance (Lundby et al., 2012). In other words, ‘excess’ vasodilatation is only ‘excess’ in terms of adequacy to support oxygen uptake, not optimization of exercising muscle metabolic and contractile function. This again leads to the implication of restraining ‘excess’ vasodilatation to remain in balance with a set $\dot{Q}$ response in submaximal exercise, which is that the $\dot{Q}$ response is calibrated to muscle metabolic demand. Yet investigations with the aim of understanding oxygen delivery–demand matching focus on the vasodilatory signals and how they are locally connected to the magnitude of oxygen demand (Clifford & Hellsten, 2004; Saltin et al., 1998). It would seem that the field of arterial blood pressure regulation in exercise and the field of exercising muscle oxygen delivery–demand matching in exercise need to integrate the contradiction of $\dot{Q}$‐driven versus exercising muscle vasodilatation‐driven circuit flow into assessment of arterial blood pressure and oxygen delivery–demand matching regulation in submaximal exercise.

If metabolic demand is communicated precisely to the controller of autonomic efferent pathways to the heart and resistance vasculature, then the basis for this communication needs to be incorporated in understanding oxygen delivery–demand matching. The observation that, as exercise intensity increases, the vasodilatation response in exercising muscle gives way to arterial pressure elevation to continue proportional increases in perfusion with metabolic demand (Gonzalez‐Alonso et al., 2008; Mortensen et al., 2005, 2013; and see Figure 3) would fit with such communication. At present, mechano‐ and metaboreceptor afferent feedback from exercising muscle is a well‐known contributor to autonomic cardiac activation and sympathetic vasoconstriction during exercise (Amann et al., 2010, 2011), but its blood pressure‐elevating effect can also act to improve exercising muscle oxygen delivery–demand matching (O'Leary & Sheriff, 1995; Sheriff et al., 1987). However, precision of communicating oxygen delivery–demand matching to achieve a calibrated $\dot{Q}$ response, as the concept of sympathetic restraint being necessary to prevent ‘excess’ vasodilatation from exceeding the set $\dot{Q}$ response to a given exercise intensity would require, would seem beyond the capability of a muscle afferent feedback pathway.

## 5 FUTURE DIRECTIONS

We have pointed out the implications of the current view that the role of sympathetic restraint is to prevent ‘excess’ vasodilatation relative to the $\dot{Q}$ response to exercise or to prevent ‘excess’ vasodilatation to avoid overperfusion of exercising muscle relative to metabolic demand. There are a number of avenues of research that would warrant pursuit.

First, studies using exercising limb adrenergic blockade during large muscle mass exercise in humans are required. Furthermore, ensuring activation of all muscles in the exercising limb would seem necessary, given that Heinonen et al. (2013) observed that non‐exercising hamstring muscle experienced vasodilatation with infusion of an adrenergic receptor blocker but exercising quadriceps muscle did not. Second, if the cardiac output response is calibrated to exercising muscle metabolic demand, then how this is achieved requires investigation, and it needs to be incorporated into our understanding of oxygen delivery–demand matching in exercise. Third, we require more measurements of the dynamic response when the balance between cardiac output and exercising muscle blood flow is disturbed. If a peripherocentric drive for cardiovascular circuit flow can achieve a steady‐state $\dot{Q}$–PBF balance without arterial blood pressure compromise, how does it get there? Fourth, we know too little about the structural characteristics of the cardiovascular circuit on the venous and pulmonary side in terms of affecting the time it takes for increased exercising muscle blood flow to appear at the left ventricle in order that systemic $\dot{Q}$ can increase accordingly. Finally, given that body position with respect to the gravity vector can alter autonomic activation and the central and peripheral cardiovascular responses to exercise (Dambrink & Wieling, 1987; Leyk et al., 1992, 1994; Mitchell & Victor, 1996), the impact of posture on the necessity for sympathetic restraint of exercising muscle vasodilatation should be considered.

Sympathetic restraint in exercising muscle exists. We would argue that its purpose and what it tells us about the nature of integrated cardiovascular circuit control in exercise are far from clear.

## AUTHOR CONTRIBUTIONS

Work on the review was conducted in the School of Kinesiology and Health Studies, Queen's University, Kingston, Ontario, Canada. Conception and design of the review, literature review, dissemination and interpretation, drafting and revising the work for intellectual content: Patrick J. Drouin and Michael E. Tschakovsky. Literature review, dissemination and interpretation, and revision of the work for important intellectual content: Stacey P. A. Forbes, Abby K. Zedic and Stuart P. S. Mladen. All authors approved the final version of the manuscript and agree to be accountable for all aspects of the work in ensuring that questions related to the accuracy or integrity of any part of the work are appropriately investigated and resolved. All persons designated as authors qualify for authorship, and all those who qualify for authorship are listed.

## CONFLICT OF INTEREST

None declared.
